# Imported malaria in the Infectious Diseases Hospital of Iaşi: epidemiological and clinical characteristics of 28 consecutive cases

**DOI:** 10.1186/1471-2334-13-S1-P83

**Published:** 2013-12-16

**Authors:** Andrei Vâță, Daniela Leca, Mihaela Cătălina Luca, Denisa Colesniuc, Cătălina Logigan, Denisa Covaciuc, Carmen Dorobăț

**Affiliations:** 1 “Gr. T. Popa” University of Medicine and Pharmacy, Iaşi, Romania; 2 Infectious Diseases Hospital Iaşi, Romania

## Background

This paper aims to describe the main characteristics of malaria cases observed in the Infectious Diseases Hospital of Iaşi in the last 16 years.

## Methods

We performed a retrospective study of the medical charts of 28 consecutive patients diagnosed with malaria between January 1998 and August 2013. The parameters analyzed were: demographical, related to the stay in the endemic country, clinical, hematological and biochemical parameters, parasitological findings, diagnosis method, treatment and prophylaxis, and days of hospital stay.

## Results

The mean annual number of cases was 1.75. The patients were aged between 18 and 60 years (mean age 32.1 years). 96.7% were males. The most frequent country of origin was Turkey (57.1%), followed by Angola (17.8%), China (7.1) and India, Ivory Coast, Ethiopia, Portugal, Equatorial Guinea – 3.6% of cases. The length of stay in the endemic country was in 82.1% of cases of more than one month. Only 21.4% of patients had received chemoprophylaxis. Fever higher than 39°C was present in all cases. Chills, myalgia, jaundice, vomiting and sometimes neurological symptoms were also present. 25% were severe forms requiring intensive care. *Plasmodium vivax* was responsible for 64.3% of cases, *P falciparum* – 28.6%, *P malariae* and *P ovale* – 3.6%. 75% of cases had anemia, 42.8% thrombocytopenia, 78.6% elevated ESR, and 28.6% renal failure. The treatment was started as soon as the diagnosis was established, 4.38 days (mean) after the disease onset. The mean hospitalization time was 11.9 days. 32.1% of patients had to be transferred to the Clinical Hospital of Infectious and Tropical Diseases “Dr. Victor Babeş”, Bucharest, due to disease’s severity, lack of medication or response to the initial anti-malarial regimen. No deaths were recorded.

## Conclusion

Although a rare disease in our area, malaria can still pose special diagnostic problems and lead to severe clinical forms in returning travelers from endemic regions.

